# Localization to, and Effects of Pbp1, Pbp4, Lsm12, Dhh1, and Pab1 on Stress Granules in *Saccharomyces cerevisiae*

**DOI:** 10.1371/journal.pone.0010006

**Published:** 2010-04-02

**Authors:** Kylie D. Swisher, Roy Parker

**Affiliations:** 1 Howard Hughes Medical Institute, University of Arizona, Tucson, Arizona, United States of America; 2 Department of Molecular and Cellular Biology, University of Arizona, Tucson, Arizona, United States of America; Victor Chang Cardiac Research Institute (VCCRI), Australia

## Abstract

The regulation of translation and mRNA degradation in eukaryotic cells involves the formation of cytoplasmic mRNP granules referred to as P-bodies and stress granules. The yeast Pbp1 protein and its mammalian ortholog, Ataxin-2, localize to stress granules and promote their formation. In *Saccharomyces cerevisiae*, Pbp1 also interacts with the Pab1, Lsm12, Pbp4, and Dhh1 proteins. In this work, we determined whether these Pbp1 interacting proteins also accumulated in stress granules and/or could affect their formation. These experiments revealed the following observations. First, the Lsm12, Pbp4, and Dhh1 proteins all accumulate in stress granules, whereas only the Dhh1 protein is a constitutive P-body component. Second, deletion or over-expression of the Pbp4 and Lsm12 proteins did not dramatically affect the formation of stress granules or P-bodies. In contrast, Pbp1 and Dhh1 over-expression inhibits cell growth, and for Dhh1, leads to the accumulation of stress granules. Finally, a strain lacking the Pab1 protein was reduced at forming stress granules, although they could still be detected. This indicates that Pab1 affects, but is not absolutely required for, stress granule formation. These observations offer new insight into the function of stress granule components with roles in stress granule assembly and mRNP regulation.

## Introduction

An important part of the cellular response to stress or environmental stimuli is the modulation of cytoplasmic mRNA translation and degradation, which allows for substantial changes in the population of pre-existing mRNAs. One aspect of this process in eukaryotic cells is the remodeling of translating mRNAs into non-translating mRNPs that accumulate in cytoplasmic foci known as P-bodies and stress granules to allow for storage and decay of mRNA. P-bodies are present in low numbers under normal cellular conditions and in elevated numbers following inhibition of translation initiation and/or many environmental stresses [reviewed in 1], [2]. Stress granules arise when translation initiation is strongly inhibited, such as during different environmental stresses or drug-induced translational repression, and contain translationally inactive mRNA and translation initiation factors [3]. Understanding the composition and formation of these two granules will provide insight into how mRNA fate is controlled during stress and during normal growth.

While the function of these large aggregates is not entirely known, their composition provides some clues as to their roles. P-bodies contain mRNA decay factors, including Dcp1/2 (decapping enzyme), Xrn1 (exonuclease), Pat1, Dhh1 and Scd6 (activators of decapping and repressors of translation), and Lsm1-7 and Edc3 (activators of decapping) [1]. Additionally, mammalian P-bodies contain components involved in miRNA silencing [1]. P-bodies also contain mRNA decay fragments, suggesting that mRNA decapping and 5′ to 3′ exonucleolytic decay can occur at these sites, although large P-bodies are not required for normal mRNA decapping in yeast [1], [4]. The composition of stress granules is different than that of P-bodies, suggesting that the two granules have different functions in the mRNA lifecycle. Stress granules contain translation initiation components and a diverse array of mRNA binding proteins, but the exact composition can vary depending upon the stress [5]–[12]. For example, during glucose deprivation, stress granules in yeast, also referred to as EGP-bodies, contain eIF4G, eIF4E, Pab1, Pub1, Ngr1, and Pbp1 [7], [8]. However, during heat shock in yeast, similar stress granules form that additionally contain eIF3, a hallmark factor in mammalian stress granules [9]. Mammalian and yeast stress granules have both been shown to contain mRNA [5], [7], [10]. The change in composition of stress granules, which is dependent upon the nature of the stress, might be explained by different stresses causing different rate-limiting steps in the assembly of translation initiation complexes [8], [9], [12].

Though P-bodies and stress granules have distinct compositions, they do not function and exist entirely separate from each other. The two granules are often juxtaposed to one another following treatment with arsenite in mammalian cells [3], [13], [14], or co-localized with each other at early time points following glucose deprivation in yeast [8]. In addition, the same species of mRNA localizes to both P-bodies and stress granules, suggesting that mRNA may transition between the two foci [3], [7]. Many mRNP components, such as mammalian Rck/p54, Xrn1, and eIF4E, can be present in both granules [3], [8], [12], [14]. Understanding how mRNA and specific proteins transition between these two granules may provide important information for further understanding the function of these granules and their effects on mRNA.

Recent studies in both yeast and mammalian cells implicate a role for Pbp1 (Pab1-binding protein) and Ataxin-2 (mammalian ortholog of Ppb1), in the assembly of stress granules, with some additional effects on mammalian P-bodies. Deletion of Pbp1 in yeast or siRNA knockdown of Ataxin-2 in mammalian cells leads to significant decreases in stress granule formation under glucose deprivation or arsenite treatment, respectively [8], [11]. In the same cells, P-body formation is not affected. Over-expression of Ataxin-2 in mammalian cells decreases P-body number in the absence of stress [11], indicating that Ataxin-2 may promote the transition of mRNPs out of P-bodies. These results suggest that Pbp1 and its interacting proteins may localize to and play roles in stress granule assembly.

Pbp1 interacts with several interesting proteins with possible roles in the control of cytoplasmic mRNA function. Pbp1 was identified by an interaction with the C-terminal domain of Pab1, and was found to exist with both the translating and non-translating pools of mRNA [15], [16]. Pbp1 also interacts with the Pbp4 and Lsm12 proteins, and these three proteins all associate with the translation machinery [17], [18]. Moreover, a physical interaction between Pbp1 and Dhh1 has been demonstrated by a protein-fragment complementation assay, as well as a physical interaction between Dhh1 and Lsm12 [19]. Given this set of interactions, we were interested in whether these factors could localize to stress granules and P-bodies, and in examining if the Pbp4, Lsm12, and Pab1 proteins played important roles in cytoplasmic RNA granule formation.

Our data suggests that the Pbp1 interacting factors, Pbp4 and Lsm12, can accumulate in stress granules, whereas Dhh1 accumulates in both P-bodies and stress granules, similar to its mammalian ortholog, Rck. Loss of Pbp4 and Lsm12, or their over-expression, did not significantly affect stress granule formation, P-body formation, or cell growth. Contrary to this, over-expression of Pbp1, Dhh1, and Pab1 caused growth inhibition. Pbp1 over-expression triggered abnormal Pab1 aggregation, and Dhh1 over-expression triggered Pab1 stress granule formation. Interestingly, Pab1 also had an effect on stress granule formation, but only in some genetic backgrounds, suggesting Pab1 has a role in stress granule formation but is not absolutely required.

## Results

### Pbp4 and Lsm12 are novel stress granule components

Pbp1 and its mammalian ortholog, Ataxin-2, accumulate in stress granules upon glucose deprivation or arsenite stress, respectively [8], [11]. In budding yeast, Pbp1 forms a complex with Pbp4 and Lsm12 and all factors associate with ribosomes [18]. These results suggest that Pbp1, Pbp4, and Lsm12 could possibly form a complex localizing in and affecting stress granule formation. To examine this possibility, we utilized chromosomally integrated GFP-tagged versions of Pbp4 and Lsm12 to see if they accumulated in stress granules or P-bodies as judged by co-localization between the GFP-marked protein and either Pub1-mCherry (stress granule marker) or Edc3-mCherry (P-body marker) during glucose deprivation (as described in materials and methods).

**Figure 1:**
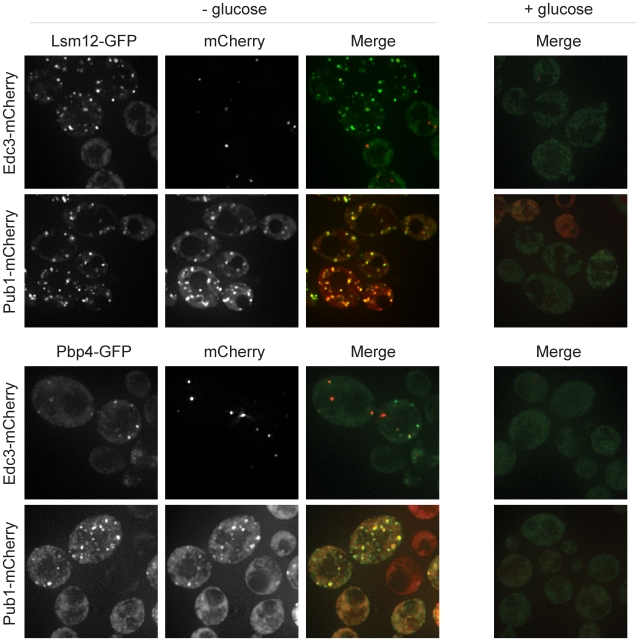
Lsm12 and Pbp4 localize to stress granules. GFP-tagged Lsm12 and Pbp4 were assessed for their ability to form granules that co-localize with either Edc3-mCherry, a P-body marker, or with Pub1-mCherry, a stress granule marker, following ten minutes of glucose deprivation stress.

Under normal conditions, Pbp4 and Lsm12 were diffuse in the cytoplasm, but following glucose deprivation stress, both accumulated in foci that co-localized with Pub1-mCherry, suggesting that they are stress granule components (Figure 1). We define stress granules as foci that 1) are not present in the absence of stress, 2) are induced upon treatment with stress, and 3) contain factors widely accepted as stress granule factors in both the yeast and mammalian fields [12]. Some Pbp4 and Lsm12 foci co-localized with the P-body marker (Edc3), but many Pbp4 and Lsm12 foci were clearly distinct from P-bodies (Figure 1). This is consistent with previous reports that show some co-localization between P-bodies and stress granules in yeast [8], [9]. We interpret these observations to indicate that Pbp4 and Lsm12 can accumulate in stress granules.

### Dhh1, a P-body component, can also localize to stress granules in yeast

Several observations suggested to us that Dhh1 might also accumulate in yeast stress granules, although it had previously only been described as a P-body component [20]. First, the mammalian ortholog of Dhh1, Rck, can accumulate in stress granules following increasing durations of arsenite stress in mammalian cells [8], [14]. Second, Dhh1 interacts with Pbp1 and Lsm12, both of which are now known to localize to stress granules, by two-hybrid and protein-fragment complementation assays [11], [19]. To examine if Dhh1 could also accumulate in stress granules as well as P-bodies, we utilized a triple fluorescent protein system where Edc3-mCherry (P-body marker) and Pab1-CFP (stress granule marker) were expressed from a plasmid in a wild-type strain containing a chromosomally integrated Dhh1-GFP.

**Figure 2:**
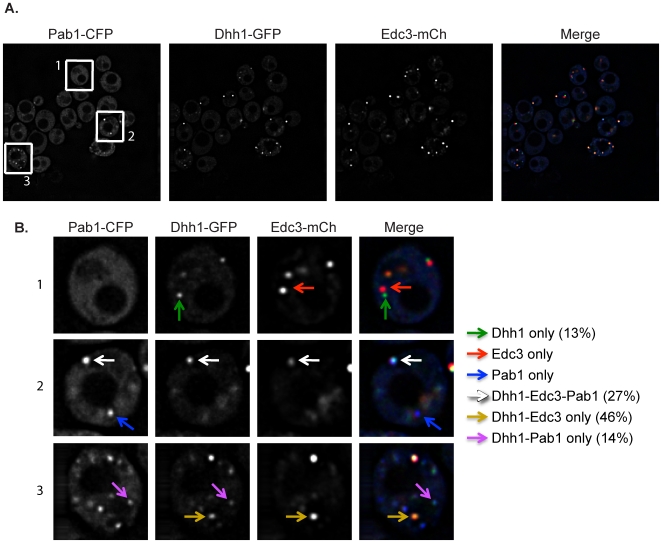
Dhh1 localizes to both P-bodies and stress granules following glucose deprivation stress. (A) A strain harboring a Dhh1-GFP integration was transformed with a plasmid containing Edc3-mCherry and Pab1-CFP and assayed for GFP co-localization with mCherry and/or CFP upon glucose deprivation. (B) A closer view of three sample cells, labeled accordingly in A, is shown. Dhh1 co-localized with Edc3-mCherry (gold arrows), Pab1-CFP (purple arrows), and both Edc3-mCherry and Pab1-CFP (white arrows). Dhh1 was also found in independent foci (green arrows).

Following glucose deprivation stress, we observed Dhh1 concentrated in clear cytoplasmic foci. The majority of these foci (73%) co-localized with Edc3, which is consistent with Dhh1 being a component of P-bodies [20]. A subset of the foci containing Edc3 and Dhh1 (27% of the total) also co-localized with Pab1 (Figure 2, white arrows), which is consistent with previous observations that stress granules and P-bodies in yeast can overlap [8], [9]. We interpret these observations to indicate that the majority of the Dhh1 foci observed during glucose deprivation are P-bodies.

Strikingly, we also observed a smaller percentage of Dhh1 foci that co-localized with Pab1 stress granules in the absence of Edc3 (14%; Figure 2, purple arrows). This indicates that while Dhh1 is predominantly in P-bodies, Dhh1 can also localize to stress granules. This is similar to what is seen in mammalian cells with the Dhh1 ortholog, Rck [8], [14].

To our surprise, we also saw some Dhh1 foci that were independent of both Pab1 and Edc3 (13%; Figure 2, green arrows). We do not yet know the identity of these Dhh1 only foci, but one possibility is that they represent P-bodies where Edc3 is predominantly replaced by Scd6, which interacts with Dhh1 in a similar and exclusive manner to Edc3 [21].

### Lsm12, Pbp4, and Pab1 are not required for stress granule formation

**Figure 3:**
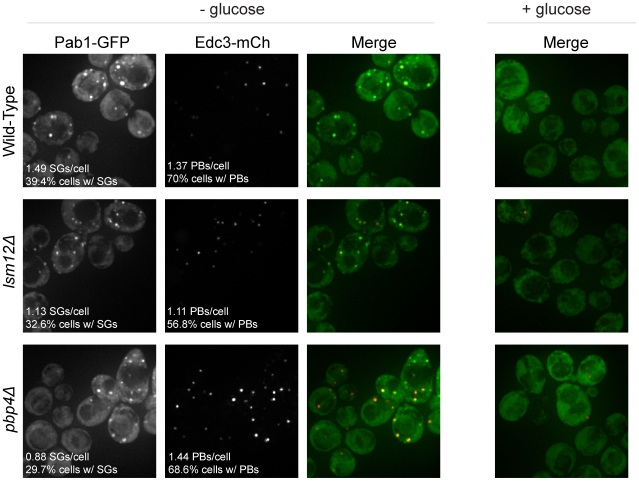
Lsm12 and Pbp4 are not required for stress granule formation. *lsm12Δ*, *pbp4Δ*, and wild-type strains were transformed with a plasmid containing Edc3-mCherry (P-body marker) and Pab1-GFP (stress granule marker) and assessed for the ability to form either granule following ten minutes of glucose deprivation stress. (SGs denotes stress granules; PBs denotes P-bodies.)

Given that the Lsm12 and Pbp4 proteins interact with Pbp1 and localize to stress granules, and that Pbp1 can affect stress granule formation [8], we wanted to determine if these proteins affected stress granule and/or P-body formation. To examine this issue, we transformed *lsm12Δ* and *pbp4Δ* strains with a plasmid expressing Pab1-GFP (stress granule marker) and Edc3-mCherry (P-body marker), and examined the formation of stress granules and P-bodies during glucose deprivation. We observed that strains lacking Lsm12 and Pbp4 were still able to form stress granules and P-bodies, although the stress granules formed at slightly reduced levels compared to wild-type (Figure 3). This indicates that these proteins are not required for stress granule formation.

We also examined if Pab1 was required for stress granule formation. *PAB1* is an essential gene in *Saccharomyces cerevisiae* and therefore to examine the role of Pab1, we utilized two strains that contain bypass suppressors of *PAB1*. The first strain is the *pab1*Δ *spb2*Δ strain, where defects in 60S subunit biogenesis lead to suppression of *pab1*Δ [22]. The second strain is the *pab1*Δ *pat1*-2 strain, which carries a nonsense mutation in the *PAT1* gene and thereby suppresses the *pab1*Δ lethality [23], [24]. To assess the ability of these strains to form P-bodies or stress granules, both *pab1Δ* suppressor strains, as well as strains harboring the individual suppressor mutations (*spb2Δ* and *pat1-2*) and the wild-type strain were transformed with a plasmid expressing Pbp1-GFP (stress granule marker) and Edc3-mCherry (P-body marker). Examination of glucose deprived cells led to the following observations.

**Figure 4:**
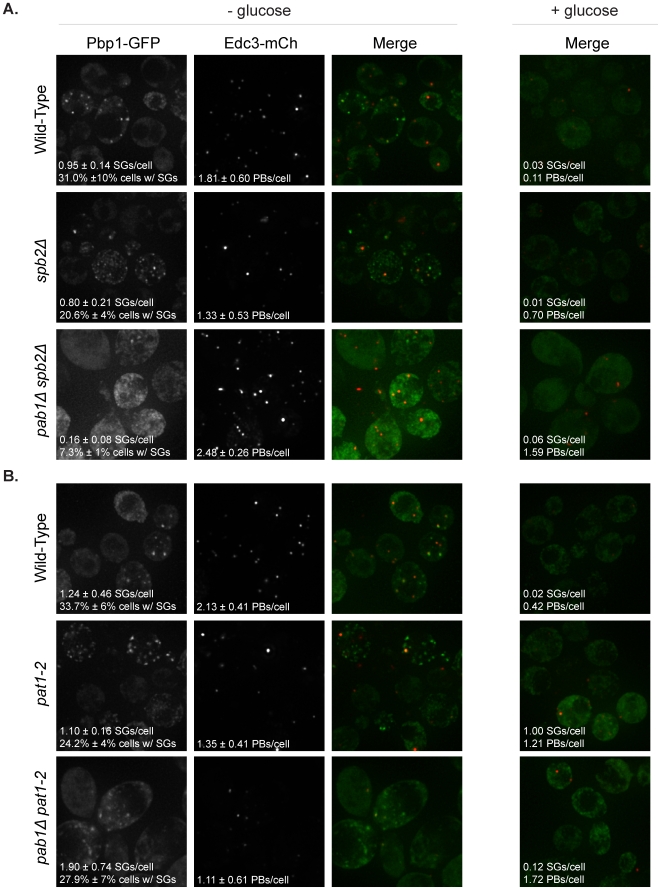
Pab1 promotes, but is not absolutely required for stress granule formation. *pab1Δ spb2Δ*, *spb2Δ*, and wild-type strains (A), as well as *pab1Δ pat1-2*, *pat1-2*, and wild-type strains (B), were transformed with a plasmid containing Pbp1-GFP (stress granule marker) and Edc3-mCherry (P-body marker) and analyzed for granule formation following ten minutes of glucose deprivation stress.

We observed that the *pab1Δ spb2Δ* strain showed an increase in the number of P-bodies per cell and a significant reduction in the number of stress granules per cell as compared to the *spb2Δ* strain alone, although low levels of stress granules were still observed (Figure 4A). We interpret this observation to suggest that Pab1 promotes stress granule formation, but is not absolutely required for stress granules to form. In addition, the corresponding increase in P-bodies in *pab1Δ spb2Δ* along with the decrease in stress granules is consistent with the previous suggestion that mRNAs primarily move from P-bodies to stress granules during glucose deprivation [8].

In contrast to the results with *pab1Δ spb2Δ,* the *pab1Δ pat1-2* suppressor strain showed a slight decrease in the number of P-bodies per cell and no change in the number of stress granules per cell (Figure 4B). This provides additional evidence that Pab1 is not absolutely required for stress granule formation. We do not yet understand why the Pab1 protein is more important for stress granule formation in the *spb2Δ* background as compared to the *pat1-2* background, but one possibility is that the loss of functional Pat1 reduces the ability of mRNAs to be maintained in P-bodies and therefore are more apt to remodel into a stress granule mRNP, even in the absence of Pab1.

Finally, and consistent with earlier results [25], we observed an increase in the number of P-bodies in the *pab1Δ* suppressor strains under normal non-stress conditions compared to the wild-type, *spb2Δ,* and *pat1-2* strains (Figure 4A and B). This is consistent with Pab1 normally functioning in part to promote mRNAs exiting P-bodies and re-entering translation.

### Over-expression of Dhh1, Pbp1, and Pab1 cause growth defects

**Figure 5:**
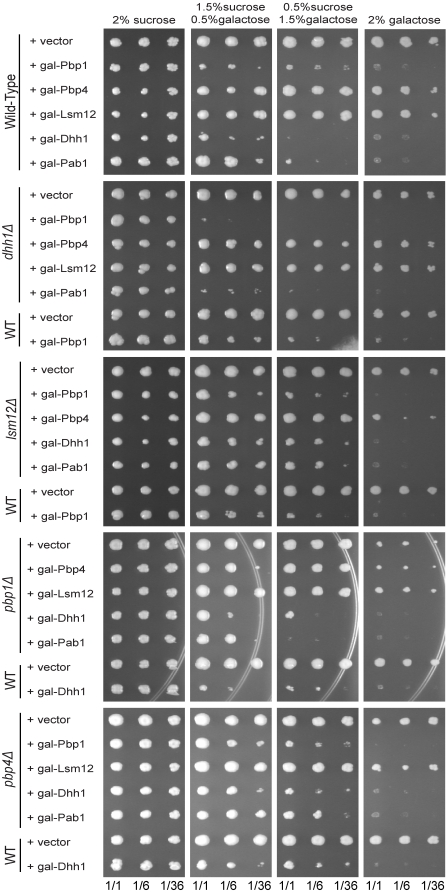
Dhh1, Pbp1, and Pab1 over-expression inhibit growth in wild-type and deletion strains. Proteins were expressed using galactose-inducible promoters on high copy plasmids and assessed for growth in both wild-type and deletion strains on media containing different concentrations of galactose as their carbon source. All plates were incubated at 30°C for five days.

Several proteins that are components of yeast or mammalian stress granules and/or P-bodies can cause growth inhibition and/or granule formation when over-expressed [reviewed in 12]. For example, over-expression of Dhh1 in yeast has been shown to inhibit cell growth [26]. Given this, we examined the effects of over-expression of Pbp1, Lsm12, Pbp4, Pab1, and Dhh1 (as a control) on cell growth and the formation of stress granules and/or P-bodies. We observed that strains over-expressing Pbp1, Dhh1, or Pab1 via a galactose-inducible promoter showed growth inhibition on plates containing 2% galactose, while strains over-expressing Lsm12 or Pbp4 grew normally (Figure 5). Dhh1 over-expression appears to cause stronger growth inhibition than Pbp1 over-expression, as assessed by growth levels on 0.5% sucrose/1.5% galactose.

### Growth inhibition phenotype caused by over-expression of Pbp1, Dhh1, and Pab1 is not suppressed by deletion of other stress granule factors

To further understand how Pbp1, Dhh1, and Pab1 over-expression might be triggering growth inhibition, we asked if specific factors were required for this phenotype by examining the over-expression inhibition of growth in strains lacking the other factors. Thus, we expressed Pbp1, Dhh1, Lsm12, Pbp4, and Pab1 under control of the galactose promoter in the *pbp1Δ*, *dhh1Δ*, *lsm12Δ*, and *pbp4Δ* deletion strains. Growth was then assessed on media containing sucrose or galactose as the carbon source.

We observed that none of the factors tested suppressed the inhibition of growth caused by Pbp1, Dhh1, or Pab1 over-expression (Figure 5). We used decreasing levels of galactose to assess whether the deletion factors had any effects on growth that could be missed due to the lack of sensitivity on media containing 2% galactose. Interestingly, we observed that in a *dhh1Δ* strain, over-expression of Pbp1 shows strong growth inhibition with lower levels of galactose induction as compared to Pbp1 over-expression in the wild-type background (Figure 5; 0.5% galactose/1.5% sucrose). Interestingly, this is similar to the previous observation that over-expression of the Stm1 protein is more toxic in a *dhh1Δ* strain [27]. This does not appear to occur in the reverse, as the growth inhibition by Dhh1 over-expression is not changed in the *pbp1Δ* strain at any galactose concentration (Figure 5). Taken together, our results suggest that the absence of Pbp1, Dhh1, Pbp4, and Lsm12 does not reproducibly suppress the growth inhibition caused by over-expression of Pbp1, Dhh1, or Pab1. Our results also suggest that Dhh1 may be antagonizing Pbp1 function, as loss of Dhh1 heightened the growth inhibition caused by over-expression of Pbp1.

### Over-expression of Dhh1 and Pbp1 induce Pab1 aggregation in the absence of stress

To further determine possible roles that Pbp1, Dhh1, Lsm12, Pbp4, and Pab1 play in stress granule formation, we asked what effect each factor has on P-body or stress granule formation following their over-expression. Since *pbp1Δ* and *dhh1Δ* strains show a loss of stress granules compared to wild-type [8], and over-expression of the mammalian ortholog of Pbp1 disrupts P-bodies [11], it is possible that the over-expression of these factors may affect granule formation and the transition of mRNA between P-bodies and stress granules. To determine this, strains expressing the Pbp1, Dhh1, Lsm12, and Pbp4 proteins under the control of the galactose promoter on high copy plasmids were assessed for their ability to form P-bodies (Edc3-mCherry marker) and stress granules (Pab1-GFP marker). To assess the effect of Pab1 over-expression on granule formation, Pbp1-GFP was used as a stress granule marker.

**Figure 6:**
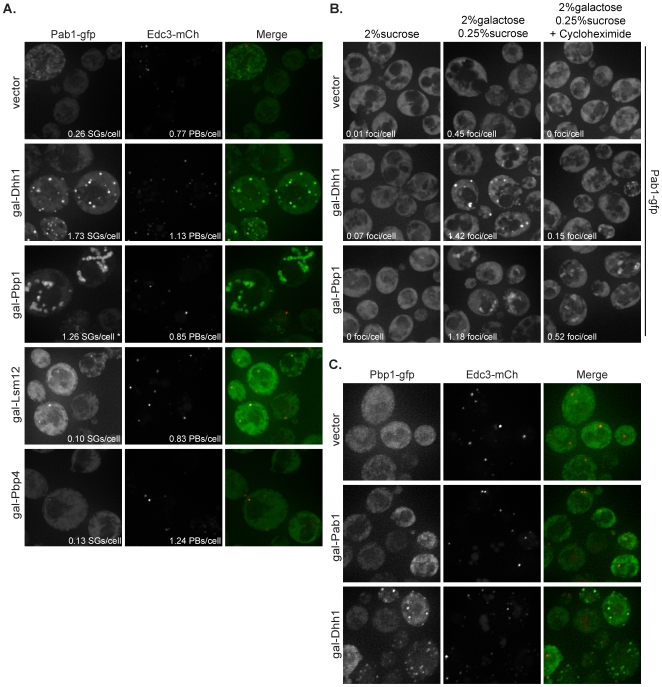
Dhh1 and Pbp1 over-expression trigger Pab1 granule formation in the absence of stress. Proteins were expressed under galactose-inducible promoters on high copy plasmids in wild-type cells. (A) Cells were assayed for their ability to form P-bodies (Edc3-mCherry) or stress granules (Pab1-GFP) in media containing 2% galactose with 0.25% sucrose. (B) Granules triggered by Dhh1 over-expression are dependent upon actively translating mRNAs. Addition of 100 µg/ml cycloheximide for 30 minutes to block translation following two hours of galactose induction substantially reduced Pab1 foci formation in cells over-expressing Dhh1, while only slightly reducing Pab1 foci in cells over-expressing Pbp1. (C) Pab1 over-expression does not cause granule formation as assessed by the P-body marker, Edc3, and the stress granule marker, Pbp1, in media containing 2% galactose with 0.25% sucrose. (*) denotes underestimate of Pab1 foci due to large string-like aggregation.

We observed that upon two hours of galactose induction and no stress treatment, strains over-expressing either Lsm12 or Pbp4 showed no increase in P-bodies or stress granules (Figure 6A), which is consistent with over-expression of these proteins causing no growth defect in yeast cells and loss of these proteins having no effect on granule formation. In contrast, over-expression of Dhh1 or Pbp1 caused substantial accumulation of Pab1 in cytoplasmic structures (Figure 6A). The Pab1 foci formed by Dhh1 over-expression morphologically resembled normal stress granules. However, the Pab1 aggregation caused by Pbp1 over-expression formed large, globular aggregates that appear to be strung together in a more fibrillar type of morphology (Figure 6A).

To determine if either of these aggregates required non-translating mRNAs for their formation, we examined how they were affected by the addition of cycloheximide, which traps mRNAs in polysomes by blocking translation elongation, depletes the pool of non-translating mRNAs, and thereby reduces stress granules and P-bodies [8], [20]. To test this, a strain containing a chromosomally integrated Pab1-GFP was transformed with either the vector control, gal-Dhh1, or gal-Pbp1 high copy plasmids. We observed that when cycloheximide was added after a two hour galactose induction, the Pab1 foci in the strains over-expressing Dhh1 declined substantially (Figure 6B), suggesting that these Pab1 aggregates are dependent upon non-translating mRNAs for their assembly. This observation argues that over-expression of Dhh1 leads to the accumulation of mRNAs in a stress granule state. In contrast, the Pab1 aggregates seen when Pbp1 was over-expressed were not as severely affected by cycloheximide addition (Figure 6B). This observation, and the aberrant morphology of these granules, suggests that the Pbp1 over-expression granules may either be less dependent upon non-translating mRNAs, be slower to disassemble, or may be protein aggregates that contain small amounts of mRNA.

The observation that Dhh1 over-expression did not lead to the accumulation of P-bodies as judged by Edc3 (Figure 6A) and Dcp2 (P-body marker; data not shown) but instead led to the accumulation of stress granules was surprising, as previous work had argued that Dhh1 over-expression led to the accumulation of P-bodies based on the subcellular distribution of Dcp2 [26]. We repeatedly saw Pab1 foci formation and no strong increase in P-body formation upon over-expression of Dhh1, allowing us to conclude that an over-abundance of Dhh1 leads to stress granule formation in the absence of stress and not P-body formation as previously reported.

We also observed that upon two hours of galactose induction and no stress treatment, strains over-expressing Pab1 showed no increase in P-bodies (Edc3-mCherry) or stress granules (Pbp1-GFP) (Figure 6C). Therefore, Pab1 over-expression triggers the same growth inhibition as Pbp1 and Dhh1 over-expression, but it leads to a significantly different granule phenotype. This result suggests that the lethality observed with excess Pab1 is not due to premature stress granules or protein aggregation.

### Pbp1 and Lsm12 are required for proper expression of other stress granule factors

Since Pbp1, Pbp4, and Lsm12 all interact and localize to stress granules, we attempted to examine whether there was a hierarchy for how these proteins assembled into stress granules. For example, we asked if Pbp4, Lsm12, and Dhh1 were able to form granules in the absence of Pbp1. To do these experiments, we crossed each of the Pbp1, Pbp4, Lsm12, and Dhh1 deletion strains with strains containing the chromosomally integrated GFP-tagged versions of the different factors (with exception of the same tagged protein as the deletion). We then determined whether the GFP-tagged factors were able to accumulate in foci in the different deletions under conditions of glucose deprivation stress. In addition, we examined the expression of each protein in the different deletion strains to determine if any of these factors were required for stable accumulation of the others. These experiments revealed the following observations.

**Figure 7:**
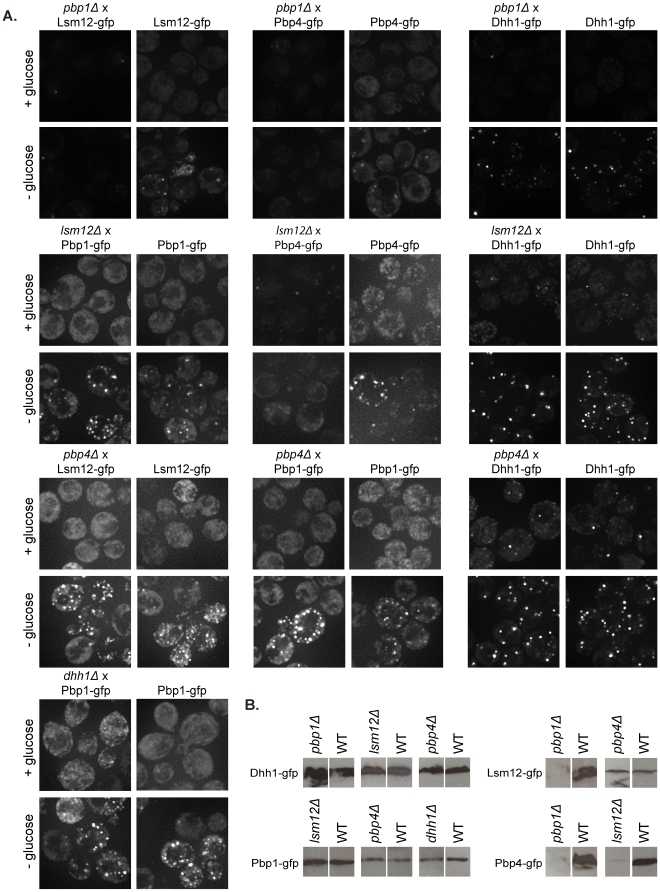
Pbp1 is required for proper expression of both Lsm12 and Pbp4. Likewise, Lsm12 is required for proper expression and granule formation of Pbp4. (A) Deletion - GFP crosses and wild-type GFP strains were assayed for foci formation following ten minutes of glucose deprivation stress. (B) Western analysis of the GFP-tagged proteins was done on whole-cell extracts using an anti-GFP antibody to detect protein levels. It is important to note that the blots have been cropped and the deletion and controls aligned with each other because, while the deletion and control strains were run on the same gel, they were not run in adjacent lanes.

First, we observed that Pbp1 is required for stable accumulation of Pbp4 and Lsm12. In the *pbp1Δ* strain, Pbp4 and Lsm12 levels were greatly reduced as judged both from the GFP signal in cells observed on the microscope and by western analysis (Figure 7A and B). Additionally, we observed that Lsm12 is required for proper expression of Pbp4, as seen by microscopy and confirmed by western analysis (Figure 7A and B). In contrast, *pbp4Δ* did not affect the expression of any factors or their accumulation in foci following glucose deprivation. Taken together, these results indicate that Pbp1 is required for the expression of Lsm12 and Pbp4, and Lsm12 is required for the expression of Pbp4, perhaps because these factors are unstable in the absence of forming a Pbp1-Pbp4-Lsm12 complex.

Second, we observed that the expression and accumulation of Pbp1 in stress granules was unaffected by *lsm12Δ*, *pbp4Δ*, or *dhh1Δ*. This indicates that Pbp1 can accumulate in stress granules independently of these interacting proteins.

Third, we observed that the levels and accumulation of Dhh1 in foci were unaffected by *pbp1Δ, lsm12Δ*, and *pbp4Δ* (Figure 7A). Moreover, triple fluorescent experiments revealed that the distribution of Dhh1 between P-bodies and stress granules was similar in wild-type and *pbp1Δ* (data not shown). We interpret this set of observations to indicate that Dhh1 can accumulate in stress granules and P-bodies independently of Pbp1, Lsm12, or Pbp4.

## Discussion

### Identification of additional components of yeast stress granules

We have identified additional components of the yeast stress granules, which include the Lsm12 and Pbp4 proteins. Our results suggest that these factors are not critical for stress granule formation, as stress granules were still able to form in their absence. One possible reason for the accumulation of Lsm12 and Pbp4 in stress granules is that they associate in a complex with Pbp1 on a specific subset of mRNAs within the stress granules and therefore play a role in the maintenance of these mRNAs in the granules, binding of important translation initiation factors to the mRNAs in these granules, or in the re-initiation process itself.

### Dhh1 can be in P-bodies *and* stress granules in yeast

Our results suggest that the translation repressor and activator of decapping, Dhh1, can exist in both P-bodies *and* stress granules following a glucose deprivation stress. This finding is consistent with similar behavior by the mammalian ortholog of Dhh1, Rck [8], [14]. Additionally, it has been observed that the *Caenorhabditis elegans* ortholog of Dhh1, Cgh-1, accumulates in P-granules during oogenesis and early developmental stages, which are mRNPs that share characteristics of stress granules [28], [29]. This provides further evidence that Dhh1 and its orthologs associate and localize to stress granules and stress-granule-like particles, as well as to P-bodies. It should be noted that because Dhh1 can be in both P-bodies and stress granules and is a DEAD-box ATPase that could help remodel the mRNP, it may play an important role in the exchange of mRNAs between these two different compartments.

It is interesting that Dhh1 and its orthologs can exist in different types of cytoplasmic granules in yeast, as this appears to be a common theme developing in the granule field [12]. The yeast translation repressor, Scd6, and its mammalian ortholog, Rap55, have been observed in both P-bodies and stress granules [Rajyaguru *et al.* submitted], [30,31]. It was observed that in the absence of stress, Rap55 co-localized with the P-body markers, Dcp1a and Ge-1, but following arsenite stress, co-localized with the mammalian stress granule marker, TIA [31]. Additionally, when the cells were permitted to recover from one hour of arsenite stress, Rap55 was now seen both in stress granules and P-bodies. These results suggest that Rap55 can shuttle between the two granules. Other factors exhibit similar localization to both granules. Xrn1, which is predominantly a P-body component, has been identified in stress granules [3], and eIF4E, which is predominantly a stress granule component, has been observed in P-bodies following stress granule induction [3], [25].

### Over-expression of Dhh1 triggers stress granule formation, not P-body formation

Our data suggests that over-expression of Dhh1 in yeast not only impairs growth, but also leads to the accumulation of Pab1 and Pbp1 (Figure 6A and C) in stress granules in the absence of stress. Furthermore, blocking mRNAs in translation by addition of cycloheximide disrupts these Dhh1 over-expression granules, suggesting that the granules are dependent upon mRNA. These results suggest that Dhh1 is shifting the balance of Pab1 from the translating pool to the non-translating pool, likely by either promoting the accumulation of Pab1 into aggregates containing both proteins and mRNA, or by heightening the levels of translation repression, thereby shifting the mRNA into non-translating pools and subsequently triggering stress granule formation. It is surprising that the over-expression of Dhh1 only affects stress granule accumulation and not P-body accumulation, contrary to what was previously reported (although conditions could differ between experiments) [26]. This suggests that excess Dhh1 may promote the quick transition of mRNA through P-bodies in order to accumulate in stress granules, or may cause the mRNA to by-pass P-bodies and directly accumulate in stress granules from the translating pool. It is possible that when stress granule formation is inhibited, in conjunction with the over-expression of Dhh1, P-body formation would now be increased, rather than stress granule formation.

An interesting question is whether Dhh1 itself localizes to these over-expression stress granules in yeast. It was previously demonstrated in mammalian cells that introduction of exogenous Rck and subsequent arsenite stress leads to the accumulation of Rck in stress granules, not P-bodies [14]. If Dhh1 is found within these over-expression stress granules, it is possible that their formation is dependent upon the Dhh1-mRNA interaction which creates a repression complex that ultimately recruits Pab1 and Pbp1. If however, Dhh1 is not found in these granules, then it may simply be triggering a stress response, thereby indirectly recruiting Pab1, or increasing translation repression to such a degree that stress granule formation is triggered.

### Pab1 is not absolutely required for stress granule formation

While Pab1 is a characteristic component of both yeast and mammalian stress granules, we have determined that Pab1 is not absolutely required for stress granule formation. Use of two different *pab1Δ* suppressor strains allowed us to observe P-body and stress granule formation in the absence of Pab1. The levels of stress granule formation were different in the two *pab1Δ* suppressor strains, as they were unchanged in the *pab1Δ pat1-2* strain compared to wild-type, and drastically reduced in the *pab1Δ spb2Δ* strain. This suggests that Pab1 may regulate the rate of stress granule formation in accordance with the presence of other factors. This raises the caveat that our results may not accurately depict the role Pab1 plays in granule formation since suppressors must be present to allow for cell viability. This is most problematic for the Pat1 mutant strain, as *pat1Δ* is known to affect stress granule and P-body formation to some extent [8], [32]. One simple possibility is that Pab1 promotes the transition of poly(A)+ mRNAs from P-bodies to stress granules, as suggested by the decrease in stress granules and increase in P-bodies in the *pab1Δ spb2Δ* strain. However, we suggest that in the *pab1Δ pat1-2* strain, the formation and/or maintenance of mRNAs in P-bodies is reduced and therefore mRNAs can accumulate in stress granules even in the absence of Pab1. Indeed, this may provide an explanation for why strains lacking Pat1 can suppress the lethality of the *pab1Δ* strain.

## Materials and Methods

### Yeast strains and growth conditions

The yeast strains used in this study, and their genotypes, are found in Table 1. All strains were grown at 30°C in synthetic complete (SC) media supplemented with the correct amino acids and containing 2% glucose, unless galactose induction was required. For galactose induction in liquid media, media was supplemented with either 2% sucrose or 2% galactose with 0.25% sucrose. For the assessment of growth upon galactose induction, strains were grown at 30°C on SC media supplemented with the correct amino acids and containing either 2% sucrose, 1.9% sucrose and 0.1% galactose, 1.5% sucrose and 0.5% galactose, 1% sucrose and 1% galactose, 0.5% sucrose and 1.5% galactose, or 2% galactose. Strains yRP2065, yRP2066, yRP2192, yRP2790, and yRP2791 were obtained from a genomic deletion library (Invitrogen/Resgen collection) and only used for assaying growth upon over-expression. Strain yRP2789 was obtained from a genomic library [33]. Strains yRP2771–yRP2774 were constructed using polymerase chain reaction (PCR) to amplify the specific gene deletion cassette from genomic library strains (Invitrogen/Resgen collection) with subsequent integration into the yRP840 background [34]. The GFP tags in strains yRP2775 - yRP2778 were constructed using PCR-based gene modification methods as previously described [35]. Yeast crosses were done by standard laboratory procedure to construct strains yRP2779–yRP2788. All constructed yeast strains were verified by PCR and all yeast strains were transformed using standard laboratory techniques. Strains yRP923 and yRP924 are described as in Table 1 [36].

**Table 1:** Yeast Strains used in this study.

Yeast Strains	Properties	References
yRP840	*MATa leu2-3,112 trp1 ura3-52 his4-539 cup1::LEU2/PGK1pG/MFA2pG*	Hatfield et al.(1996)
yRP923	*MATa leu2-3,112 trp1 ura3-52 his4-539 lys2-201 spb2::URA3 pab1::URA3*	Caponigro and Parker (1995)
yRP924	*MATa leu2-3,112 trp1 ura3-52 his4-539 lys2-201 spb2::URA3*	Caponigro and Parker (1995)
yRP1131	*MATa leu2-3,112 trp1 ura3-52 his4-539 cup1::LEU2/PGK1pG/MFA2pG pat1-2 pab1::URA3*	Hatfield et al.(1996)
yRP1134	*MATa leu2-3,112 trp1 ura3-52 his4-539 cup1::LEU2/PGK1pG/MFA2pG pat1-2*	Hatfield et al.(1996)
yRP2065	*MATa his3Δ1 leu2Δ0 met15Δ0 ura3Δ0*	Invitrogen/Resgen collection
yRP2066	*MATa his3Δ1 leu2Δ0 met15Δ0 ura3Δ0 dhh1Δ::KanMX*	Invitrogen/Resgen collection
yRP2192	*MATa his3Δ1 leu2Δ0 met15Δ0 ura3Δ0 pbp1Δ::KanMX*	Invitrogen/Resgen collection
yRP2771	*MATa leu2-3,112 trp1 ura3-52 his4-539 cup1::LEU2/PGK1pG/MFA2pG pbp1Δ::KanMX*	This study
yRP2772	*MATa leu2-3,112 trp1 ura3-52 his4-539 cup1::LEU2/PGK1pG/MFA2pG dhh1Δ::KanMX*	This study
yRP2773	*MATa leu2-3,112 trp1 ura3-52 his4-539 cup1::LEU2/PGK1pG/MFA2pG pbp4Δ::KanMX*	This study
yRP2774	*MATa leu2-3,112 trp1 ura3-52 his4-539 cup1::LEU2/PGK1pG/MFA2pG lsm12Δ::KanMX*	This study
yRP2775	*MATá leu2-3,112 trp1 ura3-52 lys2-201 cup1::LEU2/PGK1pG/MFA2pG PBP1-GFP (NEO)*	This study
yRP2776	*MATá leu2-3,112 trp1 ura3-52 lys2-201 cup1::LEU2/PGK1pG/MFA2pG DHH1-GFP (NEO)*	This study
yRP2777	*MATá leu2-3,112 trp1 ura3-52 lys2-201 cup1::LEU2/PGK1pG/MFA2pG PBP4-GFP (NEO)*	This study
yRP2778	*MATá leu2-3,112 trp1 ura3-52 lys2-201 cup1::LEU2/PGK1pG/MFA2pG LSM12-GFP (NEO)*	This study
yRP2779	*MATa leu2-3,112 trp1 ura3-52 lys2-201 cup1::LEU2/PGK1pG/MFA2pG pbp1Δ::KanMX DHH1-GFP (NEO)*	This study
yRP2780	*MATa leu2-3,112 trp1 ura3-52 lys2-201 cup1::LEU2/PGK1pG/MFA2pG pbp1Δ::KanMX PBP4-GFP (NEO)*	This study
yRP2781	*MATá leu2-3,112 trp1 ura3-52 lys2-201 cup1::LEU2/PGK1pG/MFA2pG pbp1Δ::KanMX LSM12-GFP (NEO)*	This study
yRP2782	*MATá leu2-3,112 trp1 ura3-52 lys2-201 his4-539 cup1::LEU2/PGK1pG/MFA2pG dhh1Δ::KanMX PBP1-GFP (NEO)*	This study
yRP2783	*MATá leu2-3,112 trp1 ura3-52 lys2-201 cup1::LEU2/PGK1pG/MFA2pG pbp4Δ::KanMX PBP1-GFP (NEO)*	This study
yRP2784	*MATa leu2-3,112 trp1 ura3-52 lys2-201 cup1::LEU2/PGK1pG/MFA2pG pbp4Δ::KanMX DHH1-GFP (NEO)*	This study
yRP2785	*MATá leu2-3,112 trp1 ura3-52 lys2-201 cup1::LEU2/PGK1pG/MFA2pG pbp4Δ::KanMX LSM12-GFP (NEO)*	This study
yRP2786	*MATa leu2-3,112 trp1 ura3-52 lys2-201 his4-539 cup1::LEU2/PGK1pG/MFA2pG lsm12Δ::KanMX PBP1-GFP (NEO)*	This study
yRP2787	*MATa leu2-3,112 trp1 ura3-52 cup1::LEU2/PGK1pG/MFA2pG lsm12Δ::KanMX DHH1-GFP (NEO)*	This study
yRP2788	*MATá leu2-3,112 trp1 ura3-52 lys2-201 cup1::LEU2/PGK1pG/MFA2pG lsm12Δ::KanMX PBP4-GFP (NEO)*	This study
yRP2789	*MATa leu2 ura3 his3 met15 PAB1-GFP (HIS)*	Huh et al. (2003)
yRP2790	*MATa his3Δ1 leu2Δ0 met15Δ0 ura3Δ0 pbp4Δ::KanMX*	Invitrogen/Resgen collection
yRP2791	*MATa his3Δ1 leu2Δ0 met15Δ0 ura3Δ0 lsm12Δ::KanMX*	Invitrogen/Resgen collection

### Plasmids

The plasmids used in this study are described in Table 2. Plasmid pRP1944 was constructed by NsiI digestion of Pab1 in the plasmid pRP1659, and repaired via homologous recombination using a PCR fragment containing the promoter and open reading frame (ORF) for the *PBP1* gene. Plasmids pRP1361, pRP1430, pRP1941, pRP1942, and pRP1943 were purchased from Open Biosystems [37].

**Table 2:** Plasmids used in this study.

Plasmids	Properties	References
pRP1361	gal-Dhh1; 2 µ; URA marker	Gelperin et al. (2005)*
pRP1430	gal-Pbp1; 2 µ; URA marker	Gelperin et al. (2005)*
pRP1575	Edc3-mCh; cen; TRP marker	Buchan et al. (2008)
pRP1662	Pub1-mCh; cen; TRP marker	Buchan et al. (2008)
pRP1659	Edc3-mCh; Pab1-GFP; cen TRP marker	Buchan et al. (2008)
pRP1660	Dcp2-mCh; Pab1-GFP; cen TRP marker	Buchan et al. (2008)
pRP1768	Edc3-mCh; Pab1-CFP; cen; URA marker	received from JR Buchan, U of A/HHMI
pRP1827	gal empty vector; 2 µ; URA marker	received from Elizabeth Grayhack, URMC
pRP1941	gal-Pbp4; 2 µ; URA marker	Gelperin et al. (2005)*
pRP1942	gal-Lsm12; 2 µ; URA marker	Gelperin et al. (2005)*
pRP1943	gal-Pab1; 2 µ; URA marker	Gelperin et al. (2005)*
pRP1944	Edc3-mCh; Pbp1-GFP; cen TRP marker	This study

*Purchased from Open Biosystems

### Microscopy

To perform glucose depletion and control experiments, yeast cultures were grown and assessed as described previously [8]. Briefly, cells were grown in the correct SC media in the presence of glucose to OD600 0.3–0.4. Cells were then collected by centrifugation, washed briefly in the media +/− glucose and briefly centrifuged. For glucose deprivation, cells were re-suspended in media lacking glucose and incubated in a flask at 30°C with shaking for ten minutes. Follow this, cells were collected by brief centrifugation, washed in media lacking glucose, briefly centrifuged and re-suspended for assessment by microscopy. To perform galactose induction experiments, yeast cultures were grown in the correct SC media containing 2% sucrose to OD600 0.3–0.4. Cells were then split and collected by centrifugation, washed briefly in media containing 2% sucrose or 2% galactose with 0.25% sucrose and briefly centrifuged. Cells were then re-suspended and incubated in flasks at 30°C with shaking for two hours. Following this time, cells were then collected by brief centrifugation, washed again in the appropriate media, centrifuged, and re-suspended in the appropriate media for microscopic investigation. For assessment of the effect of translation on over-expression granules, cultures were grown and shifted to galactose media, as depicted above, for two hours. 100 µg/ml cycloheximide was added for 30 minutes and cultures continued to incubate at 30°C with shaking. Cells were collected and analyzed as described above.

Images were acquired as previously described [8]. A DeltaVision RT microscope system (Applied Precision, Inc Issaquah, WA) was used with an Olympus 100×, 1.4NA objective, and the softWoRx 3.5.1 software program. Images were collected as 512×512 pixel files using a CoolSnapHQ camera (Photometrics, Tucson, AZ) using 1×1 binning. Deconvolution was done on all images using the deconvolution algorithms in the softWoRx program (enhanced ratio, low noise filtering). Image J [38] was used to adjust all images to the same contrast according to the protein being examined and the experiment. All images were taken using Z-series of 12 images and collapsed during analysis by Image J, with the exception of the triple fluorescent experiments (Dhh1-GFP, Edc3-mCherry, Pab1-CFP), which were single plane images where the CFP image was taken followed very closely by the dsRED image and the FITC image. For this experiment, to avoid bleed-through between the fluorescent GFP and CFP channels, we utilized the FITC filter to assay GFP signal. Experiments were done to verify that use of these filters removed overlap between the two channels (data not shown).

### Image quantitation

Analysis of three different individual experiments was done to quantitate the triple fluorescent datasets and assess granule co-localization. Quantitation of P-body and stress granule foci in the *pbp4Δ* and *lsm12Δ* strains was done in a blind manner for four independent datasets totaling 340–410 cells for + glucose conditions and 770–920 cells for–glucose conditions. Similar quantitation was done for the *pab1Δ* suppressor strains and their controls by analysis of three different individual experiments, but was not done blindly due to characteristic changes in cell morphology of the suppressor strains. Quantitation of Pab1 in cells over-expressing Pbp1 was done and likely under-represents the amount of Pab1 actually present in the aggregates, as the large protein formations were difficult to accurately quantitate.

### Growth Assay

To assess cell growth upon over-expression of specific factors, cultures were grown overnight in the appropriate SC media containing 2% sucrose in test tubes at 30°C with shaking. Cells were then diluted to OD600 0.1 in the same media, and incubated at 30°C for several hours until OD600 0.4. Cultures were then transferred into 96 well plates, with initial cultures at OD600 0.4. A 1/6 dilution was then made, followed by a 1/36 dilution. Each strain containing the different over-expression plasmid was repeated four times. Strains were then plated onto large agar plates containing the appropriate SC media with the range of sucrose and galactose by a 96 well pin replicator. Plates were incubated at 30°C for five days.

### Western Analysis

To assess GFP expression levels of tagged factors, western analysis of the proteins was done on whole-cell extracts from the different strains. Cultures were incubated at 30°C with shaking in the appropriate SC media with 2% glucose until OD600 0.3–0.4. The Bio-Rad protein assay was used to determine protein concentration so equal amounts of total protein were loaded on the gels. The GFP-tagged proteins were detected using an anti-GFP antibody (Covance).
